# Early MRI Versus CT Scan for Evaluation of Cerebrovascular Events in a Community Hospital: A Cost Minimization Analysis

**DOI:** 10.7759/cureus.12127

**Published:** 2020-12-17

**Authors:** Geeta Bhagia, Sanjay Kumar

**Affiliations:** 1 Internal Medicine, Benefis Health System, Great Falls, USA; 2 Internal Medicine, Monmouth Medical Center, Long Branch, USA; 3 Hospitalist, Benefis Health System, Great Falls, USA

**Keywords:** stroke, and transient ischemic attack (tia), cost

## Abstract

Background: Diffusion-weighted MRI is shown to be equally effective, as a CT scan, in diagnosing ischemic and hemorrhagic strokes. Would it be cost-effective to perform an early MRI instead of a CT head?

Methods: A retrospective chart review was conducted between October 1, 2015, through October 1, 2017, for patients admitted for possible cerebrovascular accident (CVA). Inclusion criteria were age >/= 18 years and symptoms suggestive of a stroke. Exclusion criteria were pregnancy and age <18 years. We obtained information regarding patients' length of hospital stay, imaging modalities performed, and the related cost. We performed a cost analysis by calculating the total duration and cost of hospitalization, and cost for each investigation.

Results: The study included 828 patients who underwent CT head without contrast initially. A total of 634 (76.5%) patients got MRI brain without contrast, 261 (31.5%) had MRI brain with and without contrast, 406 (49%) had magnetic resonance angiography (MRA) head without contrast, 60 patients (7.2%) had MRA neck without contrast, 272 (32.8%) had MRA neck with and without contrast, and 1 patient (0.1%) had MRA head with and without contrast. The hospital duration for all patients was 1,797 days. The average duration per patient was 1.9364 days. The total health care cost for all patients was $25,383,983. Average per patient hospitalization cost was $25,383,983/828 = $30656.98. Average per day cost for all patients would be $25383983/1.93days = $13,152,322.8. Combined costs of all MRIs performed on all patients = $1,413,014. If MRI brain with and without contrast was considered as an initial modality, a total of $335,340 can be saved on the diagnostic imaging. Ultimately, it can also help reduce the hospitalization duration.

Conclusion: Early MRI (in appropriately selected patients) can reduce the length of hospitalization and cut some health care costs. However, more studies are required to develop appropriate patient selection criteria.

## Introduction

Stroke is a leading cause of adult disability in the United States with approximately 795,000 occurrences annually and an annual cost of $34 billion [1]. Stroke is a leading cause of death worldwide, second only to ischemic heart disease [1]. Despite emerging advances in the management of a patient with ischemic stroke at many tertiary care centers, administering tissue plasminogen activator (tPA) remains the first-line modality in most of the community hospitals where endovascular procedures might not be available at an appropriate time. As per the current guidelines, the first consideration in the evaluation of patients with stroke is to rule out intracranial hemorrhage by performing a CT scan of the head without contrast which is followed by administration of intravenous tPA if the patient is a candidate and/or medical management for secondary prevention. Recent evidence suggests obtaining diffusion-weighted MRI is equally effective in diagnosing both ischemic strokes as well as hemorrhagic stroke [2]. However, as per usual practice in many community hospitals, initial CT head is followed by MRI brain which tends to prolong the overall length of hospital stay. This practice ultimately leads to increased cost and poses a patient to risks associated with hospitalization including hospital-acquired infections, delirium specially in elderly patients, unnecessary phlebotomy among others. With our study, we want to find out if performing an MRI at initial patient encounter (for suspected TIA/stroke) helps reduce the total length of hospital stay (and if it would be cost-effective to do so). 

## Materials and methods

Our study is a retrospective chart review of patients in a university-affiliated community hospital who presented to the emergency room with symptoms suggestive of stroke between October 1, 2015, through October 1, 2017, and were admitted as a case of cerebrovascular accident (CVA). Adult patients who presented to ER for symptoms suspicious of stroke/TIA and required CT head without contrast were included in the study. We obtained information regarding the date of presentation, the imaging modalities performed on admission and subsequently during the stay, timing from the presentation, length of stay in the hospital, the reason for stay, and factors prolonging the stay. Among those who required further workup were sorted out. Further workup includes, but is not limited to, MRI brain without contrast, magnetic resonance angiogram (MRA) head without contrast, and MRA neck with and without contrast. The average duration of hospital stay in order to complete the workup was calculated. Patients' total cost of hospital stay was provided by the hospital's finance department. The cost of each investigation was also obtained (Table 1), and the average cost was calculated (Table 2). This research protocol was formally reviewed and approved by the institutional review board (IRB) committee at Monmouth Medical Center (MMC) on December 14, 2017. 

**Table 1:** Cost related to hospital stay.

	Total patients	Per patient
Total length of stay	1797 days	1.93 days
Cost of stay	$25383983	$30656.98
Cost, assuming length of stay = one day	$13,152,322.8	$15,884.45

**Table 2:** Cost related to different imaging modalities. MRA: magnetic resonance angiography; w/o: without; w/: with

Test	Code	Price ($)
CT head w/o contrast	70450	405
MRI brain w/o contrast	70551	812
MRA head w/o contrast	70544	726
MRA neck w/o contrast	70547	490
MRI brain w/ and w/o contrast	70553	1075
MRA head w/ and w/o contrast	70546	1075
MRA neck w/ and w/o contrast	70549	1075

The inclusion criteria were age more than 18 years and patients presenting through MMC ER with the initial diagnosis of possible or confirmed TIA or stroke. Any pregnant females or patients less than 18 years of age were excluded from the study. 

## Results

During the specified time period, there were a total of 828 patients who were admitted for diagnosis of possible CVA, for further management. CT head w/o contrast was performed initially for all of these patients in the ER. A total of 634 (76.5%) patients got MRI brain without contrast, 261 (31.5%) underwent MRI Brain with and without contrast. MRA head without contrast was done in 406 patients (49%), MRA neck without contrast was performed in 60 (7.2%), while MRA neck with and without contrast was done on 272 (32.8%) patients. One patient (0.1%) had an MRA head with and without contrast. 

The hospital's finance department helped provide costs related to the total length of hospital stay for each patient (Table 1) as well as the number of dollars spent to complete each imaging study (Tables 2, 3). Total cost was calculated separately, and a cost-minimization analysis was performed. 

**Table 3:** Total calculated cost. MRA: magnetic resonance angiography; w/o: without; w/: with

Investigation	Cost ($) x Total patients	Total cost for each investigation ($)
CT head w/o contrast	405 x 828	335,340
MRI brain w/o contrast	812 x 634	514,808
MRI brain w/ and w/o contrast	1075 x 261	280,575
MRA head w/o contrast	726 x 406	294,756
MRA head w/ and w/o contrast	1075 x 1	1075
MRA neck w/o contrast	490 x 60	29,400
MRA neck w/ and w/o contrast	1075 x 272	292,400

Cost related to hospital stay

As seen in Table 1, the total days of hospital stay required by all of those (828) patients to complete the workup were 1797. The total cost associated with the patients’ hospital stay while waiting for getting the workup done was $25,383,983. The average length of hospital stay per patient was found to be 1797/828 =1.9364 days. The average cost of hospital stays per patient to get a complete workup done was $ 25,383,983/828= $30656.98. The average cost per patient for one day (assuming all necessary workup is completed within one day) would be $30656.98/1.93 days = $15,884.45.

Cost related to diagnostic imaging

As seen in Table 2 and Table 3, combined cost required by all 828 patients (pts) for diagnostic imaging = $1,748,354. However, if MRI brain w/ and w/o contrast was considered as an initial modality for a total of 828 patients, the total cost of diagnostic imaging would have been $1,413,014. A total of $ 335,340 could have been saved on the investigations performed. If that helps to reduce the length of hospital stay (assuming that imaging studies are completed at the earliest possible time), the cost of hospital stay can be reduced as well (as noted above). 

Based on our study we propose that performing an MRI at the initial encounter of stroke/TIA patients will benefit the hospital as well as the patients in the long run. However, we do need proper patient selection criteria and further studies are required to look into it. 

## Discussion

The choice of the initial diagnostic workup for evaluation of stroke has been re-addressed in the American Society of stroke and American Heart association 2018 stroke guidelines [3]. It is emphasized that any interventions should not delay essential immediate therapy. Hence, the choice of appropriate diagnostic investigation is of utmost importance. Non-contrast CT scan of the head is a widely used initial imaging modality. However, MRI has been shown to be more sensitive than a CT head for detection of acute ischemia and can also detect acute and chronic hemorrhages [2]. The use of diffusion-weighted imaging within 24 hours of hospitalization has shown to improve diagnostic accuracy and acts as a guide to appropriate therapy [4]. Studies have also demonstrated that specific patterns on diffusion-weighted imaging can predict stroke etiology better than conventional methods and can potentially obviate the need for echocardiography and may limit the need for further extracranial vascular imaging studies to approximately 10% [5]. Particularly, the selected number of patients who present out of the time window for tissue plasminogen therapy can still benefit from mechanical thrombectomy if they meet appropriate criteria on imaging studies including diffusion-weighted MRI or CT perfusion study (CTP) [6]. Studies have shown an overall improvement in functional outcome in such patients at 90 days after thrombectomy plus standard care compared to standard care alone [6]. Hence, in appropriately selected patients, MRI is recommended over a CT scan as the initial imaging study in order to be able to provide appropriate and timely therapy [3]. 

The cost of MRI has been weighed against the benefits it provides. There is not enough evidence to suggest the cost-effectiveness of the routine use of MRI in all patients presenting with stroke. However, guidelines do recommend the use of MRI in appropriately selected patients (inclusion criteria based on DAWN and DEFUSE3 trials) [3,7,8]. 

Our study provides a comparison of cost for individual investigations performed for most of the patients admitted for stroke evaluation and also looks into the cost of hospital stay required for those investigations to be performed. It is evident that with the appropriate selection of the patient population, an initial MRI instead of a CT head would save the hospital cost as well as improve their functional outcome (early disposition to rehabilitation facility) in the long run. 

## Conclusions

Our study suggests that at many community hospitals (including ours), the CT head at the initial evaluation of stroke often delays the further workup to the next couple of days. It prolongs the hospital stay (1.93 days per patient) and adds to the overall hospital cost for patients. However, if we have an option of performing an MRI brain (diffusion-weighted) instead of the CT head at the initial encounter, in appropriately selected patients, we might be able to improve our patients’ prognosis as well as reduce the health care cost (at least by $335,340 only on diagnostic imaging). The hospital stay can also be minimized, and early discharge to rehabilitation facilities can be possible. Further workup can be completed in outpatient settings if required. 
